# DECKO: Single-oligo, dual-CRISPR deletion of genomic elements including long non-coding RNAs

**DOI:** 10.1186/s12864-015-2086-z

**Published:** 2015-10-23

**Authors:** Estel Aparicio-Prat, Carme Arnan, Ilaria Sala, Núria Bosch, Roderic Guigó, Rory Johnson

**Affiliations:** Centre for Genomic Regulation (CRG), The Barcelona Institute of Science and Technology, Dr. Aiguader 88, 08003 Barcelona, Spain; Universitat Pompeu Fabra (UPF), Dr. Aiguader 88, 08003 Barcelona, Spain; Institut Hospital del Mar d’Investigacions Mèdiques (IMIM), Dr. Aiguader 88, 08003 Barcelona, Spain

**Keywords:** CRISPR, Genome editing, DECKO, Long non-coding RNA, lncRNA

## Abstract

**Background:**

CRISPR genome-editing technology makes it possible to quickly and cheaply delete non-protein-coding regulatory elements. We present a vector system adapted for this purpose called DECKO (Double Excision CRISPR Knockout), which applies a simple two-step cloning to generate lentiviral vectors expressing two guide RNAs (gRNAs) simultaneously. The key feature of DECKO is its use of a single 165 bp starting oligonucleotide carrying the variable sequences of both gRNAs, making it fully scalable from single-locus studies to complex library cloning.

**Results:**

We apply DECKO to deleting the promoters of one protein-coding gene and two oncogenic lncRNAs, UCA1 and the highly-expressed MALAT1, focus of many previous studies employing RNA interference approaches. DECKO successfully deleted genomic fragments ranging in size from 100 to 3000 bp in four human cell lines. Using a clone-derivation workflow lasting approximately 20 days, we obtained 9 homozygous and 17 heterozygous promoter knockouts in three human cell lines. Frequent target region inversions were observed. These clones have reductions in steady-state MALAT1 RNA levels of up to 98 % and display reduced proliferation rates.

**Conclusions:**

We present a dual CRISPR tool, DECKO, which is cloned using a single starting oligonucleotide, thereby affording simplicity and scalability to CRISPR knockout studies of non-coding genomic elements, including long non-coding RNAs.

**Electronic supplementary material:**

The online version of this article (doi:10.1186/s12864-015-2086-z) contains supplementary material, which is available to authorized users.

## Background

The recent invention of genome-editing techniques marks a change in genomics from an observational to an experimental science. By deleting, editing, activating or repressing genomic elements at will, we can test their function in a natural setting [1]. This makes it possible for the first time to comprehensively study the function of thousands of non-protein-coding genomic regulatory elements and RNAs that have been recently discovered in the human genome [2]. In this study we adapt the CRISPR approach to the targeted deletion of non-coding elements, and demonstrate its utility in the silencing of long non-coding RNA (lncRNA) genes by promoter deletion. The key advance presented here is the use of a single starting oligonucleotide for cloning a dual gRNA expression vector, conferring both convenience and scalability.

CRISPR (Clustered Regularly Interspaced Short Palindromic Repeats) is a natural bacterial immunity system, with huge potential as a simple, cheap and versatile genome editing system [3]. Repurposed as an experimental tool, the system has two components: first, a bipartite small RNA, or “guide RNA” (gRNA), designed to recognise a specific location in the genome; second, the Cas9 protein that binds the gRNA and is thereby recruited to the target site. The gRNA consists of a 20 nt targeting region that hybridises to genomic DNA upstream of a dinucleotide protospacer adjacent motif (PAM, here NGG), fused with a scaffold sequence recognised by Cas9 protein [3]. By engineering the gRNA targeting region, one may target virtually any complementary genomic locus with moderate off-target effects [4]. As an effector protein, Cas9 may either be employed as a natural nuclease [1, 5], or else catalytically inactivated and fused to a different protein domain [6]. These features make it possible to manipulate the numerous types of non-protein coding genomic elements that have so far resisted standard RNA interference (RNAi) techniques [6, 7].

The study of one gene class in particular, long non-coding RNAs (lncRNAs), has been hindered by a lack of effective loss-of-function tools [2]. Numerous yet poorly characterised, >40,000 lncRNAs have been discovered in the human genome, yet we understand the molecular function of <1 % [8]. This gap in understanding is exacerbated by the difficulty of studying lncRNA by conventional approaches. The traditional path of reverse genetic gene targeting for the entire lncRNA catalogue is not feasible [9–12]. Furthermore, doubts have been raised as to whether the observed phenotypes in knockout animals are the result of the loss of the lncRNA itself, or of an overlapping gene regulatory elements [13]. Post transcriptional RNAi approaches regularly fail for lncRNA for reasons as yet unknown, and may only be capable of acting on cytoplasmic RNA populations [14]. Moreover, RNAi is transient and often incapable of reducing targeted RNA levels beyond 50 % [15]. Finally, other elements such as enhancers or microRNA genes are not substrates for the RNAi pathway.

Another approach worthy of mention is gene silencing through integration of destabilising sequences by zinc finger nucleases. Using this method, Diederichs and colleagues managed to stably reduce levels of the MALAT1 lncRNA around 1000-fold in cell clones [16]. While effective, such approaches require a homology construct and zinc finger nuclease that is specific to each target gene, introducing an element of complexity and ruling out large-scale screening approaches.

Against this backdrop CRISPR-based deletion holds great promise as a tool for loss-of-function studies in non-coding RNAs and genomic elements [5, 17–20]. Two distinct gRNAs flanking the target region are introduced in combination with a catalytically active Cas9. The cellular non-homologous end-joining (NHEJ) mechanism repairs the resulting break [12], and in a certain proportion of cells, one or all alleles are correctly deleted. This approach is capable of removing regions from approximately 10^2^ to 10^6^ base pairs, and there is an inverse relationship between efficiency of deletion and target region size [19]. This versatility means that CRISPR deletion has been used successfully for knocking out protein-coding genes [17], enhancers [21] and microRNAs [22].

One of the most exciting applications of CRISPR is the cell-based pooled screening of many thousands of genomic elements in parallel [6, 23, 24]. This is carried out using vector pools expressing ~10,000 unique gRNA sequences, cloned using the synthesised oligonucleotide libraries of up to 200 bp [25]. Such screens require the introduction of one single viral sequence, and hence one CRISPR construct, per cell. While this is feasible for studies of protein-coding genes, where a frameshift indel caused by a single gRNA is sufficient to disrupt an entire gene [6, 23, 24], this is not the case for non-protein coding elements, which require paired gRNAs as discussed above. This introduces the need for vector systems that are capable of [1] expressing dual gRNAs from a single plasmid, and [2] are compatible with oligonucleotide library cloning. While the first condition alone has been met by a number of recent approaches [26–28], the present study describes a method that fulfills both by cloning a dual gRNA expressing plasmid using a single starting oligonucleotide.

In this study we present a CRISPR-based knockout strategy with general applicability to almost any genomic element of <1 Mb. This system, DECKO (Double Excision CRISPR Knockout), is novel for the fact it expresses dual gRNAs from a single plasmid, which is cloned using a single starting oligonucleotide. This, coupled with the lack of homology plasmid, makes the method in principle scalable from single-gene studies to high-throughput screens, while also simplifying the derivation of stable knockout cell clones. We here demonstrate the utility of this approach in studying lncRNAs by deleting the promoter of the MALAT1 lncRNA and other genes in a number of human cell lines.

## Results and discussion

### Dual excision CRISPR knockout design

**Fig. 1:**
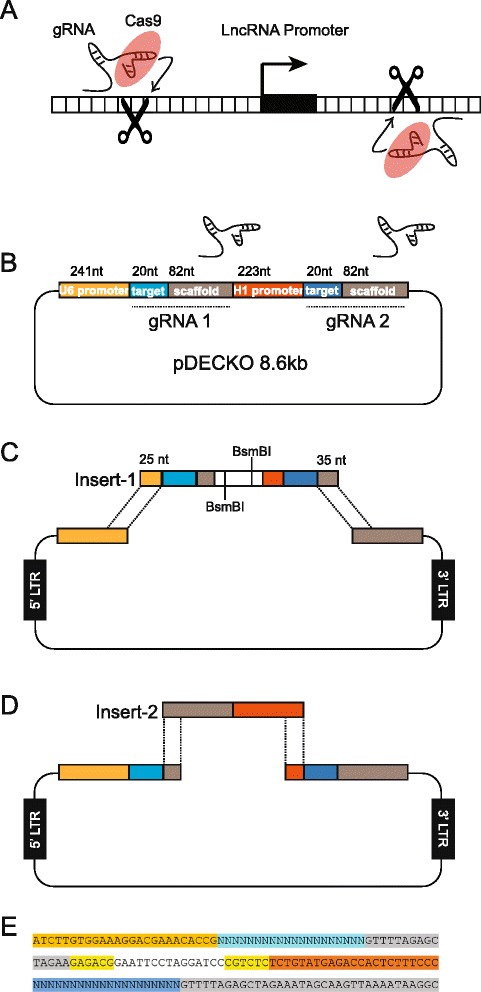
Outline of DECKO. **a** Deletion of genomic elements such as promoters by dual CRISPR targeting. **b** Structure of pDECKO. Two independent gRNA transgenes are driven by human U6 and human H1 promoter sequences. Variable sequence are highlighted in blue. **c** Cloning step 1: Synthesised oligonucleotide Insert-1, carrying all variable sequence, is inserted into BsmBI-digested parental plasmid by Gibson assembly. **d** Cloning step 2: the preassembled universal Insert-2 is inserted into the intermediate plasmid by standard ligation. **e** Nucleotide sequence of the 165 nt Insert-1. From left to right: Light orange: 5’ Gibson assembly overhang; light blue: targeting region of gRNA1; grey: fragment of first scaffold region; yellow: BsmBI sites; dark blue: target region of gRNA2; dark orange: fragment of H1 promoter; grey: 3’ Gibson assembly overhang

CRISPR can be used to delete genomic sequences, by cutting genomic DNA at two sites and relying on non-homologous end-joining (NHEJ) mechanism to repair the break (Fig. 1a). gRNAs are introduced to cells by a plasmid vector, either through transfection or viral infection. As it does not vary between experiments, the scaffold (constant region) sequence is encoded within the expression plasmid [23]. In contrast, the variable 20 nt target region must be generated in each experiment by the cloning of synthesised DNA fragment into the targeting vector. In the past, genomic deletion experiments, which require two separate gRNAs, has been achieved by co-transfecting independent plasmids, each expressing a single gRNA [5, 17, 20, 22], or else sequentially cloning two gRNA sequences into a single backbone [22]. While effective, this approach suffers from drawbacks due to the requirement for cloning two independent targeting constructs.

To facilitate the disruption of individual lncRNAs and for eventual pooled functional screens of many lncRNAs, we devised a protocol for the simultaneous cloning of two distinct guide RNA sequences into a single lentiviral vector, which we call DECKO (Dual Excision CRISPR Knock Out) (Fig. 1b). Both gRNAs’ targeting sequence is synthesised as a single 165 bp DNA fragment. The DECKO vector (hereafter, pDECKO) preparation protocol comprises two cloning stages. First, the synthesised DNA fragment “Insert-1”, containing the targeting regions of the two gRNA sequences separated by a cloning site (Fig. 1e), is inserted into BsmBI sites of parental plasmid by means of Gibson assembly cloning (Fig. 1c). As a Type IIS restriction enzyme, BsmBI cuts downstream of its recognition site, allowing scarless cloning of the gRNA sequence.

In the second cloning step, the resulting intermediate plasmid is opened at two positions by a second BsmBI digestion at the cloning sites within Insert-1, and a second DNA fragment “Insert-2” is inserted by conventional ligation, in the process removing both BsmBI sites (Fig. 1d). Insert-2 carries the gRNA constant region coupled to an H1 promoter, and does not vary between experiments. The final plasmid, suitable for transfection or lentivirus production, is capable of the independent transcription of two gRNA molecules (Fig. 1b). To avoid possible recombination or regulatory interference, gRNA genes are driven by two distinct, high-strength RNA Polymerase III promoters: human H1 and human U6 [29–32]. The entire cloning protocol lasts around 5 days, not including DNA fragment synthesis and clone validation by Sanger sequencing.

### Design of pDECKO constructs targeting lncRNA gene promoters

To test DECKO, we attempted to knock out two oncogenic long noncoding RNA genes that have been the subject of numerous previous RNAi studies: MALAT1 [16] and UCA1 [33], in addition to the protein-coding TFRC gene encoding an easily-detected surface marker protein [6].

As a general strategy for gene silencing, we chose to delete gene promoters [5]. This approach has several advantages over the conventional alternative of removing the entire gene sequence. First, it means that the region to be deleted can be in the size range 0.5–3 kb, enabling higher knockout efficiency than knockouts of 10–100 kb required for most genes [19]. Second, it means that the deleted region does not vary as a function of gene length, reducing variability between experiments. Finally, by deleting smaller regions, we can be more confident that observed phenotypic effects are due to loss of lncRNA transcription, and not an unintended consequence of deleting overlapping genomic regulatory elements [13].

**Fig. 2:**
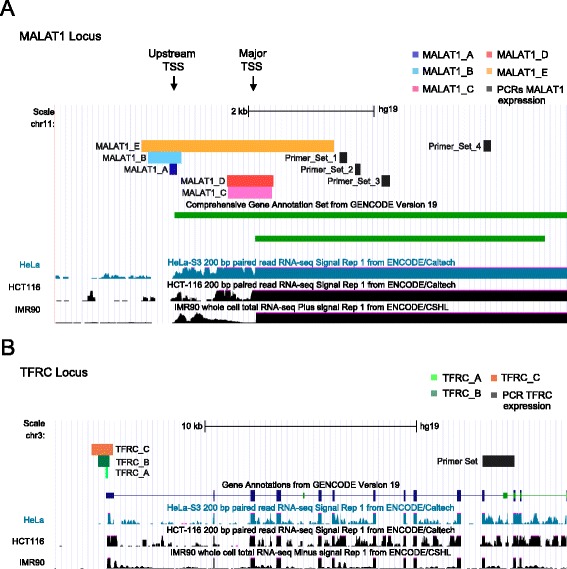
Targeting promoter regions of genes for knockout. **a** The structure of the MALAT1 locus showing the major promoter and upstream promoter. The MALAT1 transcript annotations from Gencode version 19 (*green*) lie on the positive strand. Coloured bars indicate regions targeted by CRISPR deletion in this study. Shown below are ENCODE whole cell polyA+ RNA sequencing read density for three of the cell lines used, showing that the majority of transcription originates at the major promoter. The four primer sets for qRTPCR are shown. **b** The TFRC gene locus. Coloured bars indicate regions targeted by CRISPR deletion. In black, the amplicon used for qRTPCR

We designed a series of targeting constructs (Table 1, Fig. 2 and Additional file 1: Figure S1) intended to remove varying-sized fragments encompassing the promoter and transcription start site (TSS) of the target gene set (MALAT1, TFRC and UCA1). Our selection of promoter regions was guided by both Gencode gene annotations [34] and RNA sequencing data from human cell lines from ENCODE [35]. These fragments vary in size from 100 to 3000 bp. In the case of MALAT1, we targeted both the major promoter, which seems to drive the majority of transcription, as well as a second annotated upstream promoter. Molecular cloning was carried out as described above, resulting in a full set of sequence-verified pDECKO vectors.

**Table 1:** Targeting constructs used in this study

Targeted gene	Length of cut	Position to TSS	Target region	Expected PCR size (WT)(bp)	Expected PCR size (KO)(bp)
MALAT1_A	100 bp	−70/+30	Upstream TSS	278	166
MALAT1_B	500 bp	−400/+100	Upstream TSS	641	123
MALAT1_C	600 bp	−430/+240	Major TSS	871	148
MALAT1_D	600 bp	−450/+260	Major TSS	871	109
MALAT1_E	3000 bp	−500/+2500	Both TSS	3249	224
UCA1	500 bp	−480/+160		819	181
TFRC_A	100 bp	−70/+30		215	97
TFRC_B	500 bp	−400/+100		637	141
TFRC_C	1000 bp	−700/+300		1240	239

### Expression of gRNAs from a single plasmid

**Fig. 3:**
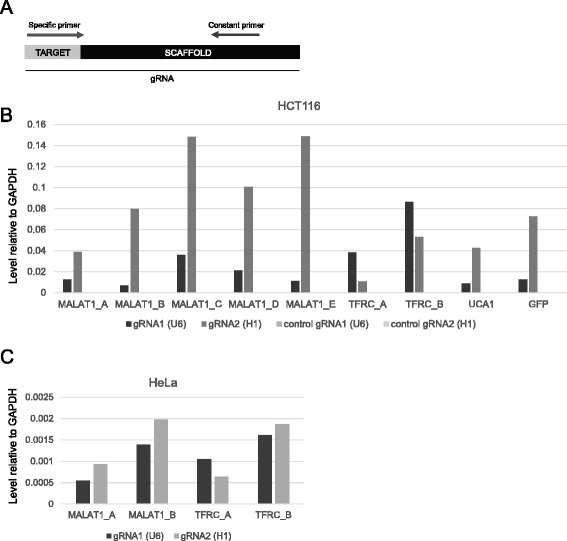
Expression of gRNAs. **a** Primer design for specific detection of gRNAs by qRTPCR. The reverse primer is constant for all PCRs while a specific primer is designed for each gRNA. (**b** and **c**) Relative expression of transfected gRNAs in HCT116 cells (**b**) and Hela cells (**c**). Cells were transfected and selected with puromycin (2ug/mL) for 3 days. Data are normalised to the housekeeping gene GAPDH. Control amplifications were carried out using indicated primers with cDNA template from pDECKO-GFP cells

We began by testing whether the pDECKO configuration is capable of expressing high levels of both gRNAs simultaneously. pDECKO plasmids were transfected into HCT116 colorectal carcinoma cells and HeLa cells, selected with puromycin and RNA was extracted. Using quantitative reverse transcriptase polymerase chain reaction (qRT-PCR) and specific primers (Fig. 3a), we observed expression for all gRNA pairs in the two human cell lines (Fig. 3b,c). Expression in HeLa (Fig. 3c) appeared more balanced between U6- and H1-driven transcripts than in HCT116 (Fig. 3b), where H1 appears to be generally the stronger promoter.

### Knockout of genomic fragments by DECKO

To examine the efficacy of pDECKO in gene silencing, we began by examining performance in bulk cells. In these experiments, although antibiotics are used to select for transfected cells, we did not attempt to isolate those cell clones where CRISPR deletion has successfully taken place. Given that (a) this cutting is a stochastic event with a defined efficiency, and (b) target site deletion depends on simultaneous cutting at two locations in one or more alleles by CRISPR, it is reasonable to expect that such bulk populations should contain a mixture of wild type, heterozygous and homozygous knockout cells.

**Fig. 4:**
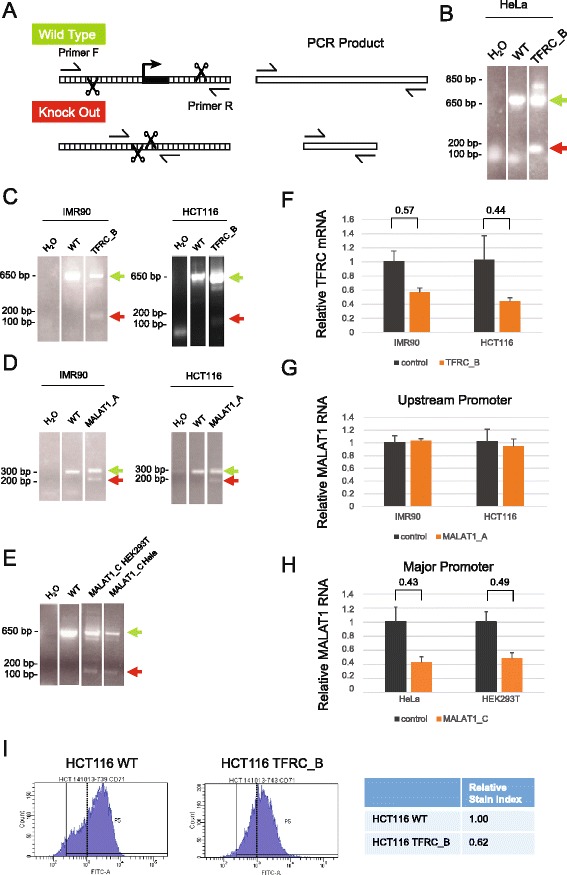
Sequence deletion in bulk cells. **a** Outline of the genomic PCR (gPCR) primer strategy used for genotyping. **b** Deletion of TFRC promoter (construct B in Fig. 2), as validated by electrophoresis of gPCR products. Wild type gDNA and water templates are used as positive and negative controls, respectively. Green and red arrows indicate the size of PCR products expected from wild type (WT) and deleted alleles. Note that in this and subsequent panels, separated lanes originate from the same original agarose gel, rearranged for clarity. **c**-**e** gPCR on bulk cells transfected with the indicated DECKO plasmids targeting (**c**) TFRC promoter, (**d**) MALAT1 upstream promoter, (**e**) MALAT1 major promoter. (**f**-**h**) qRTPCR on cell samples shown in (**c**-**e**). Control indicates RNA from cells transfected with a DECKO targeting GFP. Levels were normalised to GAPDH. Error bars show the standard deviation of three technical replicates. **i** Expression of TFRC protein on cell surface, as determined by flow cytometry analysis of antibody-stained cells. *Left*: histogram of cell fluorescence intensity counts. *Right*: Calculation of relative stain index, a normalised measure of fluorescence intensity

We transfected pDECKO plasmids into a variety of human cell lines in the presence of either cotransfected or stably-expressed catalytically-active Cas9 nuclease. Excision was tested qualitatively by genomic PCR using primers flanking the genomic target region, such that correctly deleted alleles should produce a shorter PCR product than the wild-type uncut allele (Fig. 4a). Using HeLa cells transfected with the TFRC_B pDECKO plasmid (Fig. 4b), we observed a mixture of short and long PCR products of lengths expected for knockout and wild type, respectively. These bands were confirmed to originate from the TFRC promoter by Sanger sequencing. We went on to test the TFRC_B construct in IMR90 foreskin fibroblasts, HCT116 (Fig. 4c) and HEK293T human epithelial kidney cells (Additional file 2: Figure S2A), observing in all cases short PCR products resulting from successful promoter deletion.

We next tested the effectiveness of lncRNA promoter pDECKO constructs. For vectors targeting the upstream (MALAT1_A) and major (MALAT1_C) promoters, we observed successful deletion in four different cell lines: HeLa, HEK293T, IMR90 and HCT116 (Fig. 4d and e, and Additional file 2: Figure S2B-E). We also successfully deleted the UCA1 promoter region in HEK293T cells (Additional file 2: Figure S2F). In all cases we observed a mixture of deleted and wild type alleles. Given differences in PCR amplification efficiencies and product length, it is not possible to directly infer the relative populations of these alleles given the band intensity alone. It is important to note that IMR90 are karyotypically normal and HCT116 are near diploid, while HeLa and HEK293T are aneuploid [36, 37]. HeLa cells are pseudo triploid, meaning that they are likely to carry three copies of the MALAT1 locus [36]. Altogether out of 9 DECKO plasmids tested, 8 were successful in deleting their target region in bulk cells (TFRC_A construct yielding no detectable cutting).

Promoter knockout in bulk cells should result in loss of steady-state RNA levels of the targeted genes. We tested TFRC and MALAT1 genes by qRT-PCR across all tested cell lines (Fig. 4f-h and Additional file 3: Figure S3). The steady-state levels of TFRC mRNA was reduced by around 50 % in both IMR90 and HCT116 cells (Fig. 4f). We also confirmed that this results in a loss of TFRC protein expression. Using flow cytometry analysis on cells stained with α-TFRC-FITC antibody, we observed a corresponding reduction in fluorescence of HCT116 cells transfected with TFRC_B plasmid (Fig. 4i).

Similarly we investigated the effect on MALAT1 RNA from CRISPR treatment in bulk cells. Deletion of the upstream promoter by the MALAT1_A sequences had no detectable effect on RNA levels in bulk cells (Fig. 4g). In contrast, removal of the major promoter (MALAT1_C) resulted in a clear reduction of RNA levels in both HeLa and HEK293T cells (Fig. 4h).

We observed similar results for other cell lines/pDECKO combinations, although in some cases such as TFRC_B in HEK293T we could observe no detectable reduction in target gene expression (Additional file 3: Figure S3). Thus, DECKO is capable of deleting target regions and may be optimised to achieve moderate levels of RNA knockdown in bulk cells that may be useful in some experimental contexts.

### Generation of knockout cell clones

**Fig. 5:**
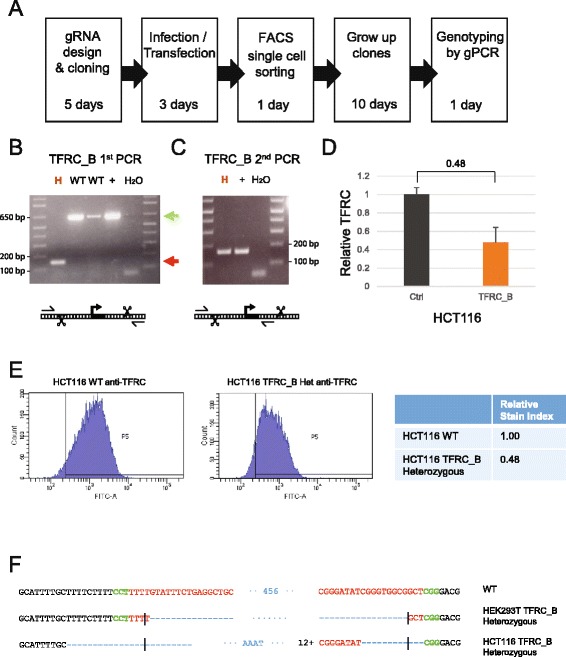
Derivation of TFRC cell clones. **a** Outline of the clone-derivation protocol used for TFRC and MALAT1 knock out cells, indicating approximate time required. **b** First and **c** second stage PCRs to genotype clones. Primer combination schemes are indicated below the electrophoresis gels. H: TFRC_B cell clone genotyped as heterozygote; WT, cell clones genotyped as wild type; +, positive control wild type cells; H_2_O, water. **d** qRTPCR for TFRC mRNA, normalised to GAPDH. Error bars indicate the standard deviation of three technical replicates. **e** Flow cytometry analysis of surface levels of TFRC protein. *Left*: histogram of cell fluorescence intensity counts. *Right*: Calculation of relative stain index. **f** Sequencing analysis of mutant junction of the heterozygous clones. In red, region complementary to the gRNA variable region; Green, PAM sequences; Blue, indel. Expected cut location is marked with vertical bar

We next sought to isolate individual cell clones carrying heterozygous or homozygous promoter deletions. Clone derivation tends to be time-consuming, given the necessity of deriving individual cell clones and genotyping them. We sought to streamline this as much as possible, through the use of FACS single cell sorting and direct PCR from cell lysates (Fig. 5a).

Cells were transfected as before with pDECKO constructs. This time, cells were separated into single clones by FACS and expanded in culture. We concentrated on HCT116, HeLa and HEK293T, since these cells could proliferate from individual clones. As before, cells were genotyped by genomic PCR, producing one of three possible outcomes: wild type cells, displaying a long PCR product only; heterozygous knock outs, producing both short and long PCR products of excised and wild type alleles; and homozygous knock outs with unique short PCR bands. We were concerned that in these experiments, the shorter knockout template might amplify more efficiently than the long, resulting in a single short band for heterozygous clones. To avoid this resulting in misidentifying heterozygous clones as homozygous, we performed an additional 2^nd^ PCR using a primer internal to the deleted region, which will only amplify wild type alleles (Fig. 5b and c). A band in the 2^nd^ PCR, coupled with a short knockout band in the 1^st^ PCR, thus indicates a heterozygous clone, as shown for the TFRC_B heterozygous clone (H) shown in Fig. 5b and c. Target fragment inversion is also a commonly observed event in experiments of this type [38, 39]. Such events will be expected to produce a wild-type-like long PCR product, possibly being misinterpreted as a wild-type allele. Therefore to identify such events, it is also necessary to genotype using an inverted internal primer (Additional file 4: Figure S4). We show below that, for the purpose of gene silencing, promoter inversion events are equivalent to deletion.

Using this combined genotyping approach, we tested the frequency of heterozygous and homozygous knockouts across 5 to 36 clones for each construct that was genotyped (Table 2), observing variable success rates. We observed higher rates of heterozygous compared to homozygous clones, (17 and 9, Table 2), out of a total of 220 tested.

**Table 2:** Frequency of clone derivation

Cell type	Target	Clones tested	WT clones	Heterozygous clones	Homozygous clones	ND
HCT116	MALAT1_A	15	10	1	4	0
HCT116	MALAT1_B	17	17	0	0	0
HCT116	TFRC_B	36	28	3	0	5
Hela	MALAT1_C	26	23	2	1	0
Hela	MALAT1_D	22	21	0	0	1
Hela	MALAT1_E	23	18	1	2	2
Hela	TFRC_B	12	12	0	0	0
HEK293T	MALAT1_C	12	10	1	1	0
HEK293T	MALAT1_D	6	3	2	1	0
HEK293T	MALAT1_E	5	5	0	0	0
HEK293T	TFRC_B	22	10	6	0	6
HEK293T	TFRC_C	24	2	1	0	21

ND – not determined

For TFRC, we first derived two heterozygous knockout clones for TFRC_B, one in HEK293T and one in HCT116, that were studied more in detail. In HCT116, qRT-PCR analysis showed that the TFRC mRNA was downregulated by 48 %, consistent with the loss of one allele (Fig. 5d). To examine the effect on protein expression, we performed flow cytometry using cells stained with α-TFRC-FITC (Fig. 5e). Consistent with mRNA results, there was an approximately 50 % decrease in TFRC immunofluorescence by this method compared to control cells, exceeding the decrease previously observed in bulk cells (Fig. 4i).

Sanger sequencing was performed on the region spanning the deleted TFRC promoter. The HEK293T heterozygous clone had the expected junction sequence of Cas9 cleavage between nucleotides 17 and 18 in the gRNA2, and one extra nucleotide on gRNA1 cut; whereas HCT116 heterozygous clone had the cutting sites 9/15 bp from the expected ones, with short fragments of inserted sequence (Fig. 5f).

In efforts to isolate TFRC homozygous knockout cells, we performed an additional clone derivation experiment using HEK293T and HCT116 cells with TFRC_B and TFRC_C DECKO constructs. Out of those 59 clones which gave rise to viable cultures, genotyping yielded a total of 8 heterozygous clones and zero homozygous clones. These results, in addition to the overall low number of viable cultures (59 out of 1152 clones sorted), leads us to tentatively propose that TFRC^−/−^ clones have reduced viability.

### Homozygous knockout of MALAT1 promoter as an effective tool for gene silencing

**Fig. 6:**
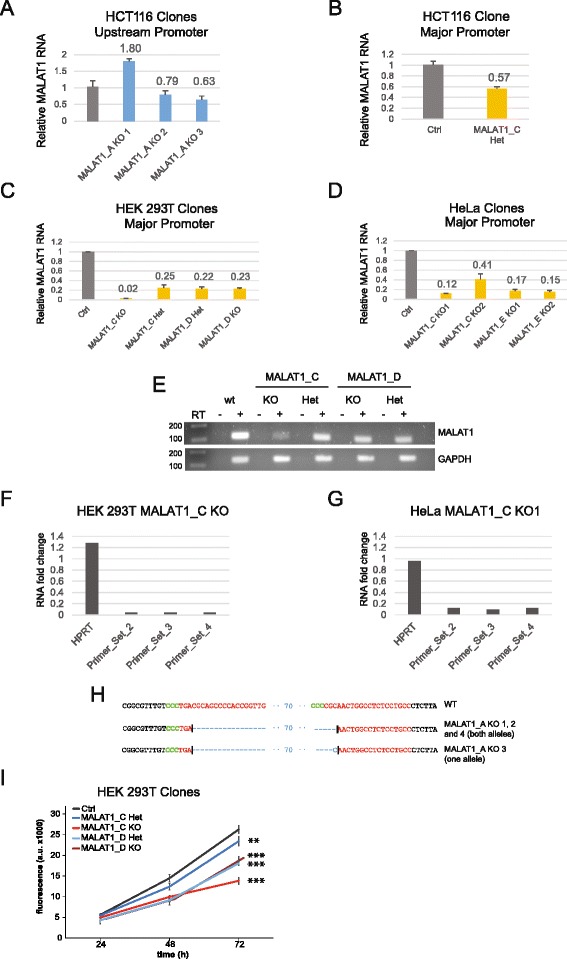
Derivation of MALAT1 knockout clones. **a** MALAT1 RNA expression levels of three HCT116 clones lacking the upstream promoter, deleted using the MALAT1_A DECKO construct. KO, homozygous knockout. Expression detected using Primer Set 1 (see below) and values are relative to a control cell clone expressing pDECKO GFP gRNAs. Error bars indicate the standard deviation of three technical replicates. **b** As for (**a**) in a heterozygous clone for the major promoter, deleted using the DECKO_C construct. Het, heterozygote. **c** MALAT1 expression in HEK293T cells heterozygous or homozygous for the major promoter, deleted using the DECKO_C & D constructs. Expression detected using Primer Set 1. **d** As for (**c**) in HeLa clones, using the DECKO_C & E constructs. **e** Semi-quantitative PCR carried out on the same HEK293T clones that in Fig. 6c. “RT-“indicates control samples where Reverse Transcriptase was omitted. **f**-**g** qRTPCR using three additional downstream primer pairs (see Fig. 2a), illustrating knockdown of MALAT1 RNA throughout the gene, for the HEK293T and HeLa MALAT1_C clones. **h** Sequence analysis of mutant junctions. MALAT1_A KO 3 contains one extra C in the deletion point. MALAT1_A 1, MALAT1_A 2 and MALAT1_A 4 have the expected deletion, with no indel. Both gRNA are in antisense direction. Expected cut location is marked with vertical bar. **i** Proliferation assay comparing the growth rates of control GFP and MALAT1 HEK293T knockout and heterozygous clones. Error bars indicate standard deviation of five cell wells. **, *P* < 0.01/***, *P* < 0.001 by Student’s one-sided, paired *t* test

We next derived a series of MALAT1 knockout clones with the same strategy described for TFRC (Fig. 5a), obtaining a total of 10 heterozygous and 9 homozygous clones in HCT116, HeLa and HEK293T (Table 2 and Fig. 6a-d). These clones were genotyped by three-step PCR as described for TFRC above. We sought to confirm that deletion of MALAT1 major promoter resulted in expected loss of steady state RNA levels in knockout clones (Fig. 6b-d), as we previously observed in bulk cells. Using primers in the body of the gene, downstream of both promoters, we performed qRT-PCR across the range of cell clones with heterozygous (Het) and homozygous (KO) deletion of MALAT1 promoter.

Similar to results in bulk cells, clones lacking the upstream promoter region of MALAT1 do not display a strong reduction in RNA levels (Fig. 6a). None of the KO clones achieved 50 % reduction in RNA levels and one clone displayed a moderate increase (MALAT1_A KO1). This latter may reflect natural epigenetic variability amongst cell clones, since two other similar knockout clones (MALAT1_A KO2,3) show a weak reduction in RNA levels (Fig. 6a). Similar variability amongst knockout clones has been observed previously [16].

Clones lacking the major promoter of MALAT1 display stronger effects on expression. The single heterozygous promoter deletion of the C construct that we obtained in HCT116 cells resulted in RNA levels 57 % of wild type (Fig. 6b). In HEK293T, heterozygous and KO cells yielded reductions of MALAT1 RNA to between 25 and 2 % of control cells (Fig. 6c), and of between 41 and 12 % in HeLa (Fig. 6d). These rates are comparable to or exceed the performance of siRNAs, although not competitive with destabilisation approaches utilising homologous recombination [16]. Clones with heterozygous promoter knockouts did have reductions comparable to some full knock outs (Fig. 6c). Similar results were observed using semi-quantitative RTPCR (Fig. 6e). These reductions in expression were also observed using three additional primer sets at downstream locations in the MALAT1 gene (Fig. 6f, g and Additional file 5: Figure S5).

We used Sanger sequencing to examine at single-base resolution the mutated sites in these cells, where necessary using TA cloning to isolate individual alleles. For MALAT1_A KO clones, three had the expected cut in both alleles, while one clone had the expected cut in one allele and a single indel in the other (Fig. 6h). For MALAT1_C and D, all clones had the expected cutting site at both alleles except for HEK293T MALAT1_D KO clone, which contained 100 bp of extra sequence between target sites (data not shown).

MALAT1 is a potent proto-oncogene, and reduction in MALAT1 RNA leads slowing of proliferation in a wide range of transformed cells [40]. We used cell growth as a phenotype by which to gauge the effectiveness of DECKO in reducing MALAT1 activity. Homozygous and heterozygous pMALAT HEK293T clones were tested over 72 h in standard proliferation assays (Fig. 6i). These cells displayed a clearly lower growth rate compared to control cells treated, starting at 24 h post-seeding. MALAT1_C KO cells, with the greatest loss of MALAT1 expression (Fig. 6c), show the most severe reduction in proliferation, with approximately half the cellular population at 72 h compared to the control clone (pDECKO-GFP).

## Conclusions

We have presented DECKO, a new strategy for the derivation of knockout clones for essentially any non-coding genomic element, including long non-coding RNAs. The key feature of the system are [1] its delivery of two distinct gRNAs from the same viral vector, [2] the synthesis of all variable sequence in a single DNA fragment at the initial cloning phase, [3] its lack of requirement of a homology construct. Together these features make DECKO not only practical for knockout of individual sites, but for screening approaches that require multiplex cloning of many target constructs in a pooled format [23, 41, 42]. The array-based megasynthesis required to produce such oligonucleotide libraries are capable of generating pools at the 165 bp length required by DECKO [23, 25, 43].

DECKO was specifically designed with the knockout of long non-coding RNAs in mind. LncRNA are likely to include many thousands of genes with roles in fundamental biological and disease processes [17]. Unfortunately progress in testing these genes’ functions at the experimental level has been hampered by the technical difficulty in manipulating them. We show that CRISPR in general, and DECKO specifically, can be used effectively to knock down lncRNA expression, paving the way for comprehensive functional testing. What is more, the versatility of CRISPR means that it should be possible to delete individual exons, or even fragments of exons of lncRNAs, allowing us to experimentally dissect the functionality of these genes at a level that has been impossible until now.

DECKO was effective in deriving knockout cell lines: 8/9 pDECKO constructs cut their intended target region in bulk studies. We designed a clone-selection workflow based on cell-sorting and direct genomic PCR to derive and genotype knockout clones with relatively low hands-on time. Altogether we managed to derive 9 homozygous knockout clones, representing 4 distinct CRISPR targeting constructs in 3 different cell lines. These homozygous clones were derived at a rate of between 1/26 and 4/15 clones tested for MALAT1. Heterozygous knockout clones were derived at a slightly higher rate: 17 individual clones from nine construct/cell combinations, with a maximum frequency of 2/6 clones tested. These knockout clones had the desired phenotypic effects: RNA levels were reduced to between 2 and 41 % of control levels, resulting in a reduction of cellular proliferation, a key MALAT1 phenotype. Together these findings show that DECKO is a practical way of knocking out lncRNAs, within reach of a typical molecular biology laboratory, and there remains scope for further efficiency improvements in future.

CRISPR genome editing enables us to directly observe the effect of genomic perturbations of gene regulatory elements. In the case of MALAT1, we have shown that CRISPR can be used to dissect gene regulatory sequences by demonstrating that the annotated upstream promoter of MALAT1 is largely irrelevant for correct gene expression in the cells used here.

While DECKO was designed with future pooled screening experiments in mind, a number of technical considerations remain. First, pooled screening requires the delivery of a single viral particle per cell. In future experiments it will be necessary to demonstrate that DECKO functions efficiently when expressed from a single genomic integration, as opposed to multiple copies of transfected plasmid. This is related to the general challenge of improving CRISPR efficiency, which is presently hampered by the large size of the Cas9 protein and our limited understanding of optimal gRNA targeting [44]. A second consideration relates to annotation of target regions: lncRNA promoters are often poorly annotated [2], and a suitable DECKO library design strategy will have to flexible enough to maximise the likelihood of knocking out the correct region.

## Methods

### Targeting sequence design

gRNA sequences were designed with the CRISPR Design tool from MIT (http://crispr.mit.edu/). U6-driven gRNAs were required to start with a G, while H1-driven gRNAs could start with any nucleoside. The highest scoring gRNAs within a window of 200 bp were chosen (Additional file 6: Table S1).

### Design and cloning of plasmids

Insert-1 sequences were designed by combining the two designed target sequences with simple design template (available as Additional file 7: File S1). These were synthesised as DNA oligonucleotides (gBlock, IDT) of 165 bp at a concentration of 20 ng/ul, and cloned using Gibson assembly method [45] into lenti-guide puro plasmid (Addgene ref. 52963) [24] digested with BsmBI (Thermo Fisher). We mixed 20 ng of Insert-1 with 100–150 ng of BsmBI-digested plasmid in 10 ul volume, with 10 ul of 2x Gibson mix (note that this step could also be carried out using commercially-available Gibson assembly reagents). We incubated the mixture at 50 °C for 1 h, and fast transformed 2 ul of this into 50 ul of z-Stbl3 competent cells (prepared with Mix and Go *E.coli* Transformation Kit, Zymo Research). The resulting intermediate plasmid, that contained additional internal BsmBI sites, was digested with BsmBI and dephosphorylated with alkaline phosphatase (Thermo Fisher EF0654). The Insert-2 sequence was previously assembled from four oligonucleotides (IDT) (Additional file 6: Table S2). These were annealed by mixing each oligo at a final concentration of 10 uM in 10 ul final volume, denatured for 5’ at 95 °C and cooled to 25 °C at a ramp rate of 0.1 °C per second. Then, a ligation reaction was performed with 50 ng of BsmBI-digested intermediate plasmid, 1 ul of annealed Insert-2 (diluted 1:20) and 1 ul of Quick ligase (Biolabs) and incubated 10 min at room temperature. 5 ul of the ligation product was transformed into 50ul of z-Stbl3 competent cells. Clones were tested by colony PCR and by Sanger sequencing using primer sequences found in Additional file 6: Table S3.

### Cell lines and culture

All cells were grown in Dulbecco’s modified Eagle’s medium (DMEM, Life Technologies), except for IMR90 that were grown in Eagle’s Minimum Essential Medium (EMEM, ATCC). Media were supplemented with 10 % fetal bovine serum (FBS, Gibco), 5 % Penicillin-Streptomycin Streptomycin (Life Technologies). Cells were maintained at 37 °C in a humid atmosphere containing 5 % CO_2_ and 95 % air. Cells were transfected with Lipofectamine 2000 (Life Technologies) following the manufacturer’s protocol. For lentivirus production, pDECKO plasmid was co-transfected into HEK293T cells with the packaging plasmids pVsVg (AddGene 8484) and psPAX2 (Addgene 12260).

For creating the Cas9 stable expressing cell lines, we transfected Cas9 plasmid with blasticidin resistance (Addgene 52962) and selected for more than 5 days with blasticidin (4ug/ml for HeLa cells and 10ug/ml for IMR90 cells).

When cotransfecting the Cas9 plasmid (Addgene 52962) together with the gRNA plasmid (pDECKO, that contains puromycin resistance), we selected for at least 2 days with puromycin (2ug/ml).

### Clone derivation and genotyping

Cells were sorted for single cell in FACS Aria or FACS Influx and plated in 96 well plates, then cultured for approximately 10 days until sufficiently numerous for genotyping. Surviving clones were transferred to 24 well plates when appropriate. For genotyping, cells were pelleted and resuspended with 50ul of Lysis Buffer (25 mM NaOH, 0.2 mM EDTA) and heated at 95 °C for 30 min [46]. The reaction was inactivated with Tris Buffer (40 mM Tris–HCl) and lysates centrifuged for 5 min at 4000 rpm. 5ul of the supernatant was used directly as a template for genomic PCR. For long amplicons such as the MALAT1_E genotyping, the PCR with this method did not work, and we extracted gDNA with GeneJET Genomic DNA Purification Kit (Thermo Scientific). A first PCR was done with primers flanking the deleted region (Additional file 6: Table S4) as shown in Fig. 4a. The absence of the wild type allele was reconfirmed with a second PCR (primers in Additional file 6: Table S5) that only amplifies WT allele as shown in Fig. 5c. In order to identify inverted alleles, we performed a final inversion PCR reaction using one external and one inverted internal primer, as shown in Additional file 4: Figure S4A (primers in Additional file 6: Table S6).

### TA cloning

In order to sequence mutated alleles in homozygous clones, we amplified junctions by PCR and cloned the resulting PCR products by TA cloning (Life Technologies), according to manufacturer’s instructions, and sequenced by Sanger sequencing using the manufacturer’s provided sequencing primer.

### RNA extraction, reverse transcription and qPCR

RNA extractions from 5 × 10^5^-2 × 10^6^ cells were performed with Quick RNA Miniprep Kit (Zymo Research). DNAse treatment was performed on-column as indicated. 150 ng-1ug RNA were retrotranscribed with Reverse Aid reverse transcriptase (Life Technologies). In all cases, a control reaction was prepared without RTase (“no RT”) in order to detect genomic DNA contamination. Quantitative PCR (qPCR) was performed with SYBR Green master mix (Roche) and in LightCycler^R^480 Real-Time PCR System (Roche). Primer sequences can be found in Additional file 6: Table S7 and S8. All quantifications were normalized to an endogenous control (Hipoxanthine-guanine phosphoribosyl transferase, HPRT; or Glyderaldehyde 3-phosphate dehydrogenase, GAPDH). The relative quantification value for each target gene compared with the calibrator is expressed as 2^(Ct-Cc). Products from the qPCRs were loaded in 2 % agarose gels to check for correct size.

### Cell proliferation assay

Cells were seeded at a density of 10^3^ cells per well in NUNC™ MicroWell™ 96-Well Microplates. Cell proliferation was monitored at 24 h, 48 h and 72 h incubating cells with 0.4 mM Resazurin sodium salt (Sigma), dissolved in PBS, at 37 °C for 2.5 h. Resazurin fluorescence was measured with an Infinite 200 PRO series multiplate reader (TECAN) using 530 nm excitation and 590 nm emission wavelengths.

### Flow cytometry

Cells were trypsinized, pelleted and washed once with PBS. Fc receptor blocker was used in order to prevent nonspecific antibody binding. Cells were incubated with α-human CD71 FITC (Bioscience, 11–0719) for 30 min in the dark (note that CD71 is an alternative designation for TFRC). Cells were rinsed with PBS + 3%FBS and DAPI was added for marking cell viability. For the stain index calculation we used the formula: (mean positive – mean background) / (2 * SD background), as previously described in [47].

## Additional files

Additional file 1: Figure S1. The targeted region of the UCA1 gene. The UCA1 gene locus, indicating the targeted promoter region. (PDF 349 kb) Additional file 2: Figure S2. Genomic PCR of bulk cells transfected with pDECKO constructs. (A) The deletion of TFRC_B promoter in HEK293T cells is shown, (B) MALAT1_A in HEK293T cells and (C) HeLa cells, (D) MALAT1_E promoter in HCT116 cells, (E) MALAT1_E promoter in HEK293T and HeLa cells, and (F) UCA1 promoter in HEK293T cells. Wild type gDNA and water are used as positive and negative controls, respectively. Green and red arrows indicate the size of PCR products expected from wild type and deleted alleles, respectively. (PDF 469 kb) Additional file 3: Figure S3. qRTPCR of targeted genes in bulk cells. (A) HEK293T cells with TFRC_B pDECKO, (B) with MALAT1_A pDECKO or (C) HeLa cells with TFRC_B pDECKO, (D) with MALAT1_A pDECKO,(E) with MALAT1_E in HeLa cells and (F) with MALAT1_E in HEK293T cells. Levels were normalised to the HPRT gene expression (A-D) or to GAPDH (E-F). Control indicates clonal pDECKO-GFP cells. (PDF 210 kb) Additional file 4: Figure S4 Inverted PCRs for genotyping. (A) Diagram of the genotyping primers configuration for detecting target site inversions. Upper image: wild type; lower image: inversion. (B). Example of PCR amplification of inverted fragment in HEK293T clones. MALAT1_D KO was determined as non-inverted clone, while MALAT1_C KO has an inversion. (C) Example of PCR amplification of inverted fragment in HeLa clones. WT HeLa was used as negative control of the PCR. (PDF 23682 kb) Additional file 5: Figure S5. qRTPCR of MALAT1 with downstream primers, complete data. RNA expression level is shown for MALAT1 clones in HEK293T and HeLa cells using different MALAT primer sets (1 to 4) (see Fig. 2) and primers for HPRT. Levels were normalised to GADPH. (PDF 286 kb) Additional file 6: Table S1. gRNA targeting sequences. **Table S2.** Sequences used to create Insert-2. **Table S3.** Sequencing primers for pDECKO. **Table S4.** Genotyping primers (1^st^ PCR). **Table S5.** Genotyping primers (2^nd^ PCR). **Table S6.** Genotyping primers (inversion PCR). **Table S7.** Primers for gRNA detection. **Table S8.** qRTPCR primers. (DOCX 22 kb) Additional file 7: File S1. Design tool for Insert-1 oligonucleotides. (XLSX 6 kb)
