# Erratum to: ‘DECKO: Single-oligo, dual-CRISPR deletion of genomic elements including long non-coding RNAs’

**DOI:** 10.1186/s12864-016-2544-2

**Published:** 2016-03-09

**Authors:** Estel Aparicio-Prat, Carme Arnan, Ilaria Sala, Núria Bosch, Roderic Guigó, Rory Johnson

**Affiliations:** Centre for Genomic Regulation (CRG), The Barcelona Institute of Science and Technology, Dr. Aiguader 88, 08003 Barcelona, Spain; Universitat Pompeu Fabra (UPF), Dr. Aiguader 88, 08003 Barcelona, Spain; Institut Hospital del Mar d’ Investigacions Mèdiques (IMIM), Dr. Aiguader 88, 08003 Barcelona, Spain

Unfortunately, the original version of this article [1] contained an error. In the Methods part, in the Design and Cloning of Plasmids section, a sentence was included incorrectly. The correct sentence can be found below

"The Insert-2 sequence was previously assembled from four 5'-phosphorilated oligonucleotides (IDT)".

Please also note in table S3 The oligo pDECKO_seq_R is lacking one nucleotide. The correct sequence is ATG**T**CTACTATTCTTTCCCC
