# Supplementary figures and images for: DECKO: Single-oligo, dual-CRISPR deletion of genomic elements including long non-coding RNAs
Source: BMC Genomics. 2015 Oct 23;16:846. doi: 10.1186/s12864-015-2086-z (PMC4619085; doi:10.1186/s12864-015-2086-z)

# FIGURE S1

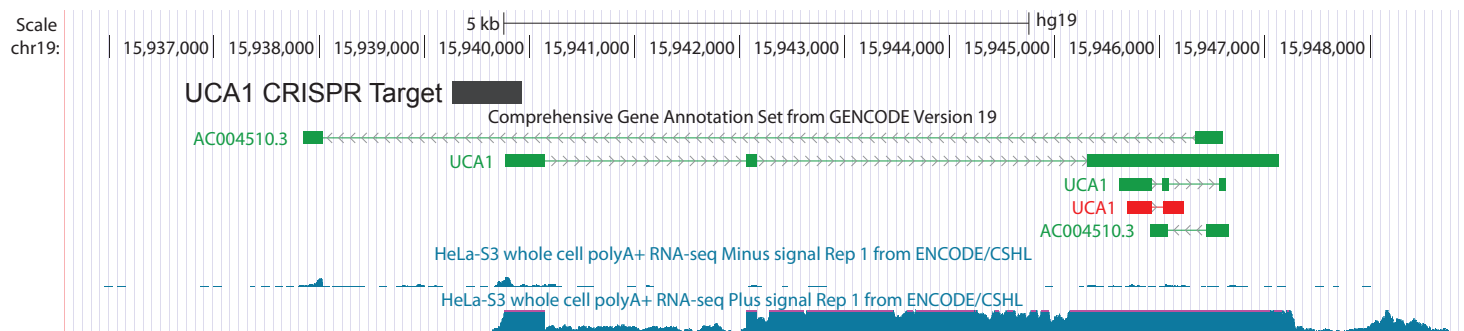

Supplement: Additional file 1: Figure S1. — The targeted region of the UCA1 gene. The UCA1 gene locus, indicating the targeted promoter region. (PDF 349 kb) [file 12864_2015_2086_MOESM1_ESM.pdf]

FIGURE S2

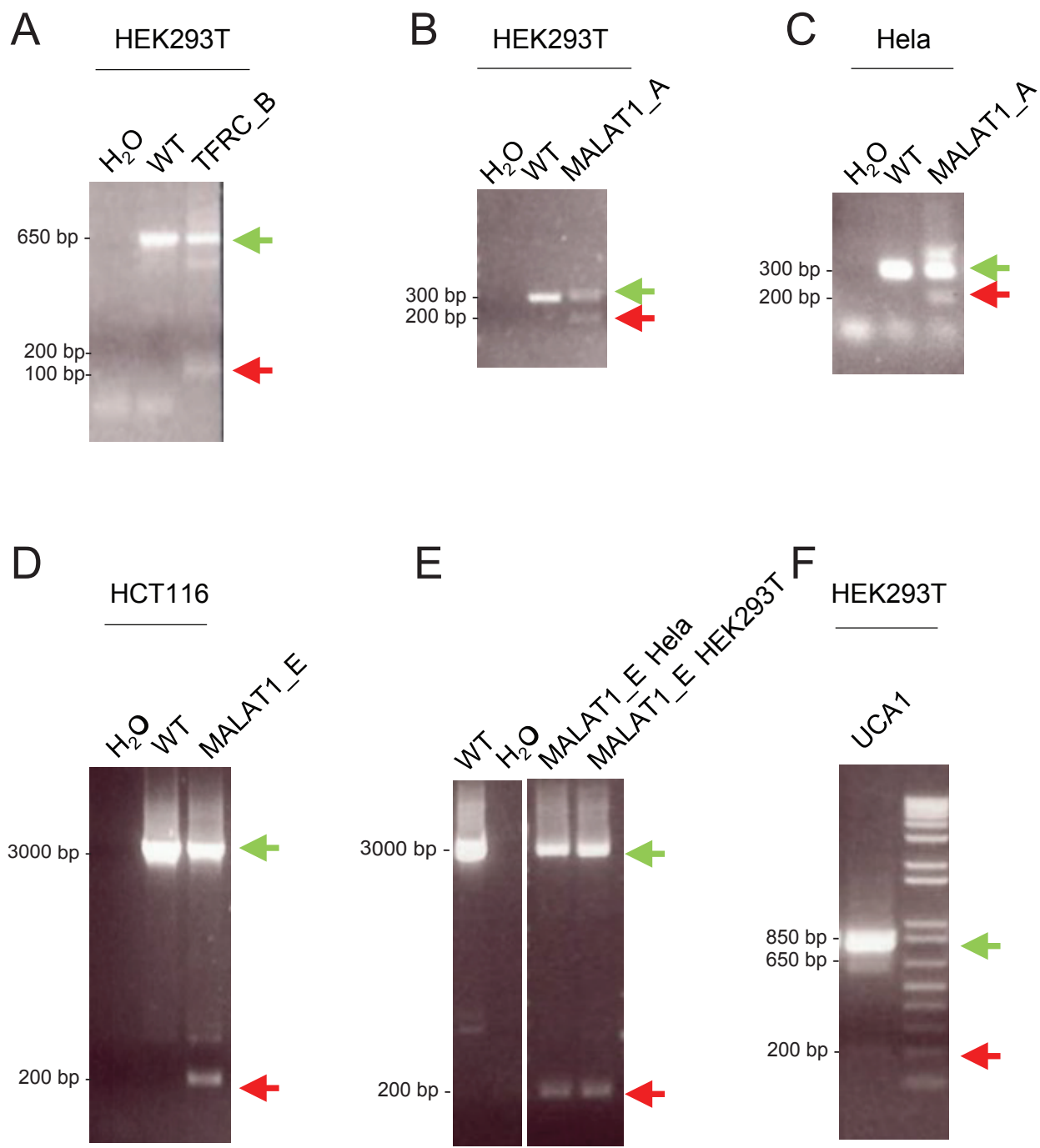

Supplement: Additional file 2: Figure S2. — Genomic PCR of bulk cells transfected with pDECKO constructs. (A) The deletion of TFRC_B promoter in HEK293T cells is shown, (B) MALAT1_A in HEK293T cells and (C) HeLa cells, (D) MALAT1_E promoter in HCT116 cells, (E) MALAT1_E promoter in HEK293T and HeLa cells, and (F) UCA1 promoter in HEK293T cells. Wild type gDNA and water are used as positive and negative controls, respectively. Green and red arrows indicate the size of PCR products expected from wild type and deleted alleles, respectively. (PDF 469 kb) [file 12864_2015_2086_MOESM2_ESM.pdf]

# FIGURE S3

## A

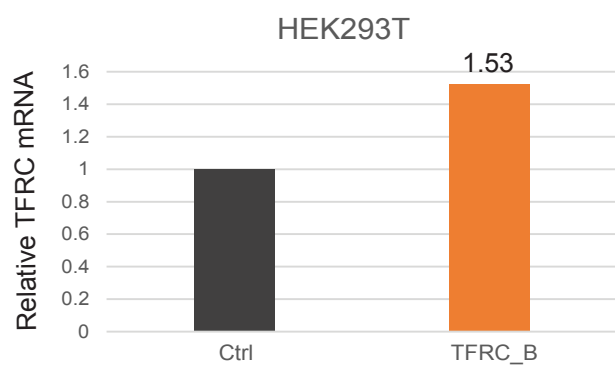

## B

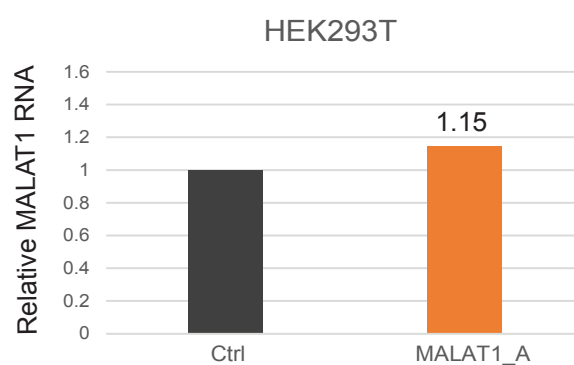

## C

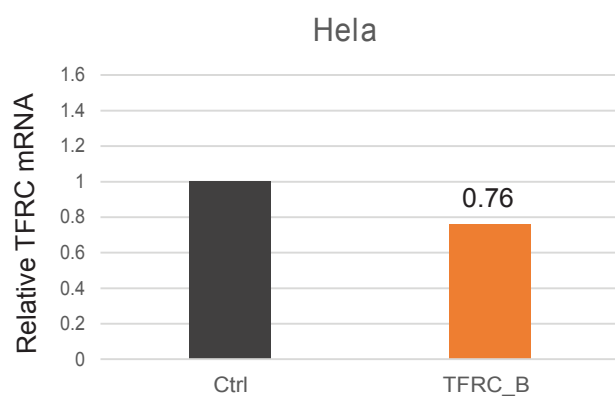

## D

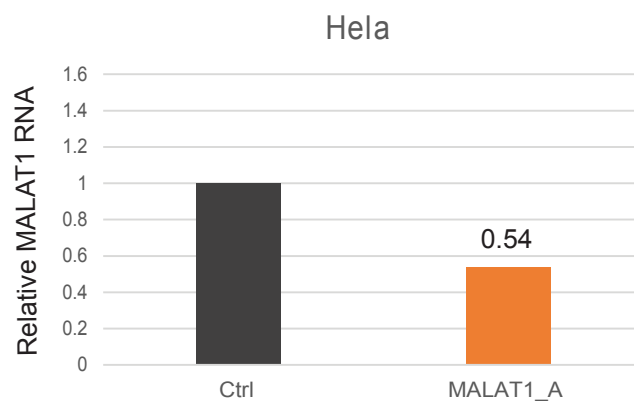

## E

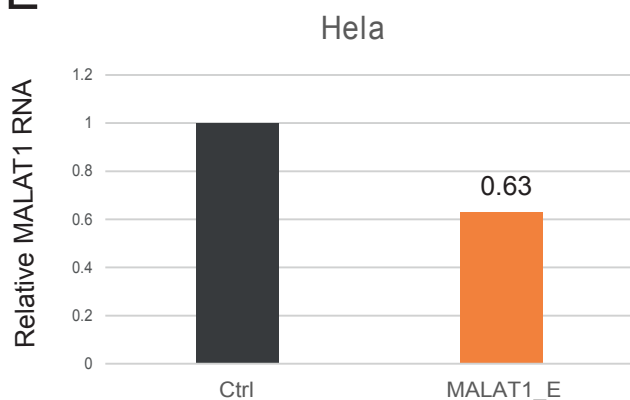

## F

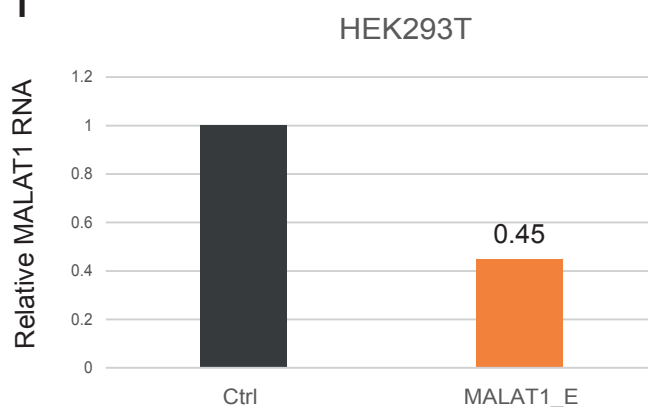

Supplement: Additional file 3: Figure S3. — qRTPCR of targeted genes in bulk cells. (A) HEK293T cells with TFRC_B pDECKO, (B) with MALAT1_A pDECKO or (C) HeLa cells with TFRC_B pDECKO, (D) with MALAT1_A pDECKO,(E) with MALAT1_E in HeLa cells and (F) with MALAT1_E in HEK293T cells. Levels were normalised to the HPRT gene expression (A-D) or to GAPDH (E-F). Control indicates clonal pDECKO-GFP cells. (PDF 210 kb) [file 12864_2015_2086_MOESM3_ESM.pdf]

FIGURE S4

A

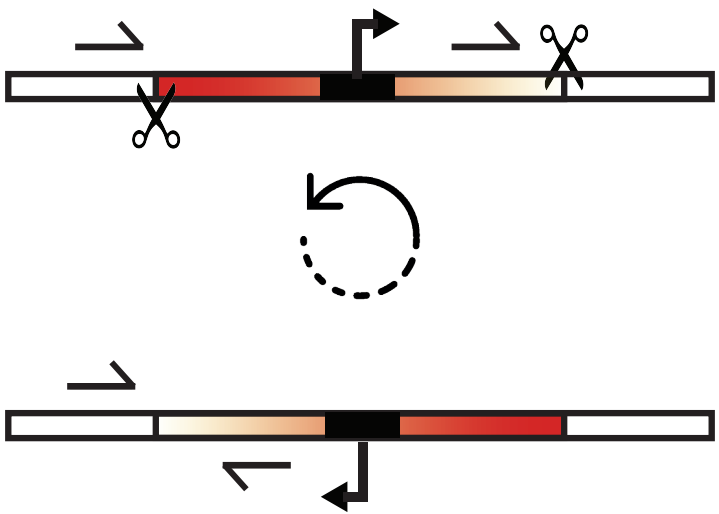

B

HEK 293T

MALAT1\_D KO  
MALAT1\_C KO

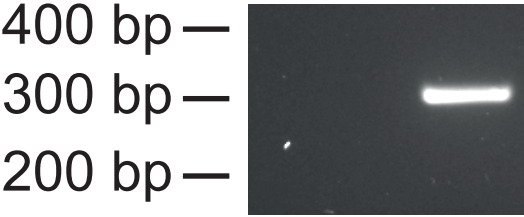

C

HeLa

MALAT1\_C KO1  
Ctrl

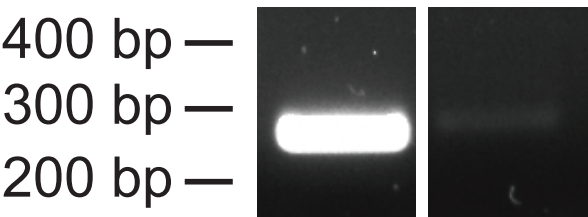

Supplement: Additional file 4: Figure S4 — Inverted PCRs for genotyping. (A) Diagram of the genotyping primers configuration for detecting target site inversions. Upper image: wild type; lower image: inversion. (B). Example of PCR amplification of inverted fragment in HEK293T clones. MALAT1_D KO was determined as non-inverted clone, while MALAT1_C KO has an inversion. (C) Example of PCR amplification of inverted fragment in HeLa clones. WT HeLa was used as negative control of the PCR. (PDF 23682 kb) [file 12864_2015_2086_MOESM4_ESM.pdf]

FIGURE S5

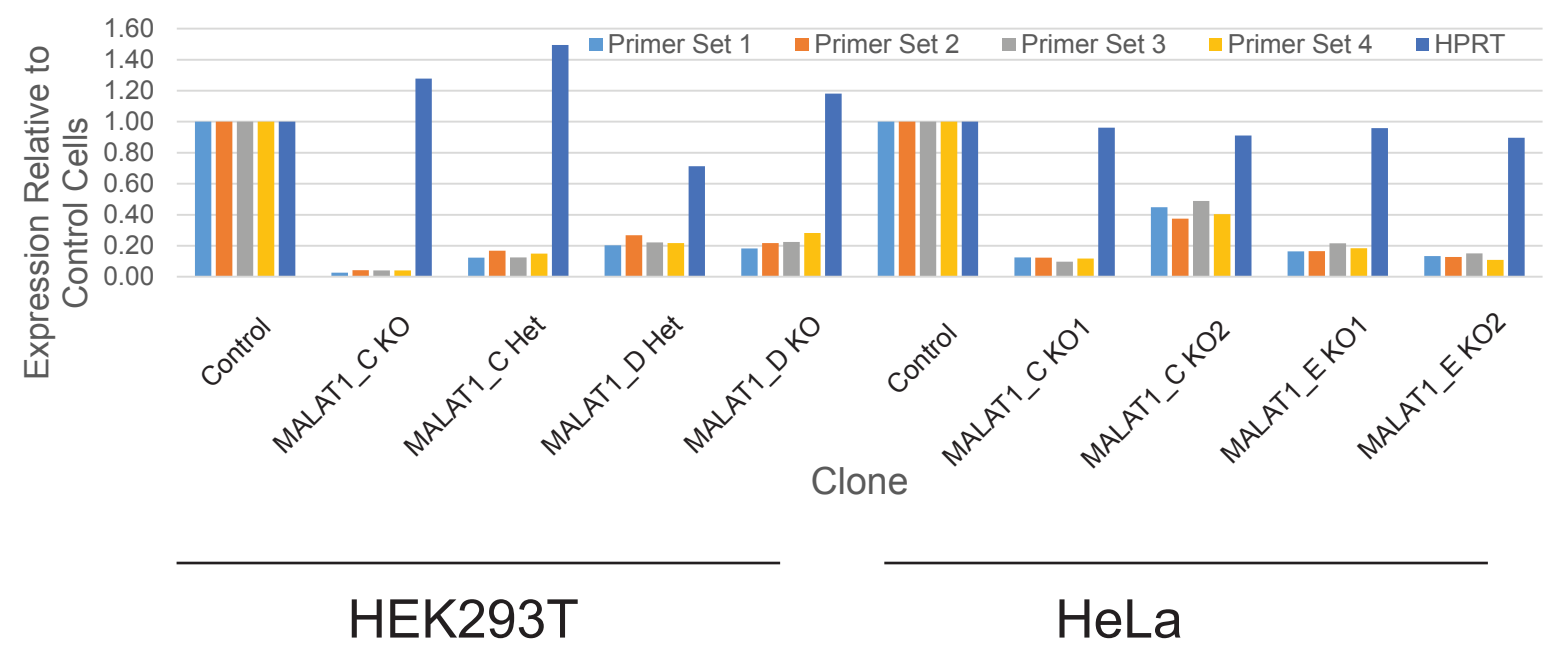

Supplement: Additional file 5: Figure S5. — qRTPCR of MALAT1 with downstream primers, complete data. RNA expression level is shown for MALAT1 clones in HEK293T and HeLa cells using different MALAT primer sets (1 to 4) (see Fig. 2) and primers for HPRT. Levels were normalised to GADPH. (PDF 286 kb) [file 12864_2015_2086_MOESM5_ESM.pdf]
