# Supplementary material for: DECKO: Single-oligo, dual-CRISPR deletion of genomic elements including long non-coding RNAs
Source: BMC Genomics. 2015 Oct 23;16:846. doi: 10.1186/s12864-015-2086-z (PMC4619085; doi:10.1186/s12864-015-2086-z)
Supplement: Additional file 6: Table S1. — gRNA targeting sequences. Table S2. Sequences used to create Insert-2. Table S3. Sequencing primers for pDECKO. Table S4. Genotyping primers (1st PCR). Table S5. Genotyping primers (2nd PCR). Table S6. Genotyping primers (inversion PCR). Table S7. Primers for gRNA detection. Table S8. qRTPCR primers. (DOCX 22 kb) [file 12864_2015_2086_MOESM6_ESM.docx]

| **Targeted gene** | **Sequence targeted** | **gRNA1 (U6 promoter)** | **gRNA2 (H1 promoter)** |
| --- | --- | --- | --- |
| MALAT1_A | -70/+30 | GAACCGGTGGGGCTGCGTCA | GGCAGGAGAGGCCAGTTGCG |
| MALAT1_B | -400/+100 | GCAACTTCCATTTTCAGTCT | GGAAGCCTCAGCTCGCCTGA |
| MALAT1_C | -430/+240 | GCTGGGGCTCAGTTGCGTAA | AGGTTTCTAAAACATGACGG |
| MALAT1_D | -450/+260 (+260 as gRNA1) | GTTGAGATGAAGCTTCTTCA | TCAACCGTCCCTGCAAGGCT |
| MALAT1_E | -500/+2500 | GAAACCTCGTGTAGCTATCA | AATGTGAAGGACTTTCGTAA |
| UCA1 | -480/+160 | GGTTTCCTTTTAGATGACGG | TCTGAAAAGAGAGTCAGCGA |
| TFRC_A | -70/+30 | GGGATGCGCGCGCAGCGGGC | TTATAGCCTGGCCCCGCCCC |
| TFRC_B | -400/+100 | GCAGCCTCAGAAATACAAAA | CGGGATATCGGGTGGCGGCT |
| TFRC_C | -700/+300 (+300 as gRNA1) | GGGGAGCGGGAAAGCGGTCG | AACTGACCTTCAGGCCCGTA |
| GFP | +60/+560 | GAGCTGGACGGCGACGTAAA | CAGAACACCCCCATCGGCGA |

**Table S1: gRNA targeting sequences.**

**Table S2: Sequences used to create Insert-2.**

| Forward_1 | TAGAAATAGCAAGTTAAAATAAGGCTAGTCCGTTATCAACTTGAAAAAGTGGCACCGAGTCGGTGCTTTTTTGAACGCTGACGTCATCAACCCGCTCCAAGGAATCGCGGGCCCAGTGTCACTAG |
| --- | --- |
| Forward_2 | GCGGGAACACCCAGCGCGCGTGCGCCCTGGCAGGAAGATGGCTGTGAGGGACAGGGGAGTGGCGCCCTGCAATATTTGCATGTCGCTATGTGTTCTGGGAAATCACCATAAACGTGAAATGTCTTTGGATTTGGGAGTCTTATAAGTT |
| Reverse_1 | GCGCACGCGCGCTGGGTGTTCCCGCCTAGTGACACTGGGCCCGCGATTCCTTGGAGCGGGTTGATGACGTCAGCGTTCAAAAAAGCACCGACTCGGTGCCACTTTTTCAAGTTGATAACGGACTAGCCTTATTTTAACTTGCTATT |
| Reverse_2 | ACAGAACTTATAAGACTCCCAAATCCAAAGACATTTCACGTTTATGGTGATTTCCCAGAACACATAGCGACATGCAAATATTGCAGGGCGCCACTCCCCTGTCCCTCACAGCCATCTTCCTGCCAGG |

**Table S3: Sequencing primers for pDECKO.**

| Primer name | sequence |
| --- | --- |
| pDECKO_seq_F | GTACAAAATACGTGACGTAG |
| pDECKO_seq_R | ATGCTACTATTCTTTCCCC |

**Table S4: Genotyping primers (1^st^ PCR).**

| **Name** | **Sequence** |
| --- | --- |
| MALAT1_A F | CGGCTAGAGCCGGTTAGAAC |
| MALAT1_A R | GGGCTTCTGCGTTGCTAAA |
| MALAT1_B F | GGAGGAAATGACAAAGGACAGG |
| MALAT1_B R | ATAGCAGCACAACTCGTCGC |
| MALAT1_C F and MALAT1_D F | GGGAGCAAGTCGCAGGA |
| MALAT1_C R and MALAT1_D R | AAAAGCATTGCCCTTCTATTGG |
| MALAT1_E F | GCCTGAGACCACTTCTGCC |
| MALAT1_E R | TGGGTCAGCTGTCAATTAATGC |
| TFRC_A F | GCCTTTTCCCTTGGCCTT |
| TFRC_A R | CGATATCCCGACGCTCTGA |
| TFRC_B F | CGCAAAGCACTCCGCTAGT |
| TFRC_B R | ACACGAGGGTCGGTGTAGTTC |
| TFRC_C_F | CCAAGTACTTTCTCTATCTTAGAGC |
| TFRC_C_R | CAGAGAGAAGGGAAGGGAC |
| UCA1 F | CTGACCAGAGAGGTATTTCCAAGA |
| UCA1 R | CCAAGTGTCAAGCATGTCCG |

**Table S5: Genotyping primers (2^nd^ PCR).**

| **Name** | **Sequence** |
| --- | --- |
| MALAT1_A F | CGGCTAGAGCCGGTTAGAAC |
| MALAT1_A R 2nd | CAGAAGTCTCGGGCTGCA |
| MALAT1_B F | GGAGGAAATGACAAAGGACAGG |
| MALAT1_B R 2nd | CTGCGCTGTCACTGCACTT |
| MALAT1_C F and MALAT1_D F | GGGAGCAAGTCGCAGGA |
| MALAT1_C R 2nd and MALAT1_D R 2nd | CACTTCTTGTGTTCTCTTGAGGG |
| MALAT1_E F | GCCTGAGACCACTTCTGCC |
| MALAT1_E R 2nd | GGCAGAAGGCTTTTGGAAGA |
| TFRC_A F | GCCTTTTCCCTTGGCCTT |
| TFRC_A R 2nd | CTCGCGAGGTGCTCTGAC |
| TFRC_B F | CGCAAAGCACTCCGCTAGT |
| TFRC_B R 2nd | GAAATGTACGTGCAGGATGGA |
| TFRC_C F | CCAAGTACTTTCTCTATCTTAGAGC |
| TFRC_C R 2nd | CAAACAATCACACCCTCTC |
| UCA1 F | CTGACCAGAGAGGTATTTCCAAGA |
| UCA1 R 2nd | CAGAGGACAGCCTGAGATGTG |

**Table S6: Genotyping primers (inversion PCR).**

| **Name** | **Sequence** |
| --- | --- |
| MALAT1_C F and MALAT1_D F | GGGAGCAAGTCGCAGGA |
| MALAT1_C invers R and MALAT1_D invers R | GGCGGAGCTTGAGGAAACC |
| MALAT1_E invers F | CCTGTCCTTTGTCATTTCCTCC |
| MALAT1_E R | TGGGTCAGCTGTCAATTAATGC |
| TFRC_B F | CGCAAAGCACTCCGCTAGT |
| TFRC_B invers R | GTCAGAGCACCTCGCGAG |
| TFRC_C invers F | GAACTACACCGACCCTCGTGT |
| TFRC_C R | AGGGACGCCTCTGCGCACAG |
| UCA1 invers F | TGTAACTTTCCACAGCCTACCC |
| UCA1 R | CCAAGTGTCAAGCATGTCCG |

**Table S7: Primers for gRNA detection.**

| **Name** | **Sequence** |
| --- | --- |
| Common gRNA expression R | GACTCGGTGCCACTTTTTCAAG |
| MALAT1_A gRNA1 | GTGGGGCTGCGTCAGTTT |
| MALAT1_A gRNA2 | CAGGAGAGGCCAGTTGCG |
| MALAT1_B gRNA1 | GCAACTTCCATTTTCAGTCTGTTTT |
| MALAT1_B gRNA2 | AAGCCTCAGCTCGCCTGA |
| MALAT1_C gRNA1 | CTGGGGCTCAGTTGCGTAA |
| MALAT1_C gRNA2 | AGGTTTCTAAAACATGACGGGTTT |
| MALAT1_D gRNA1 | CAACCGTCCCTGCAAGG |
| MALAT1_D gRNA2 | GAGATGAAGCTTCTTCAGTTTTAGAGC |
| MALAT1_E gRNA1 | CCTCGTGTAGCTATCAGTTTTAGAGC |
| MALAT1_E gRNA2 | GTGAAGGACTTTCGTAAGTTTTAGAGC |
| TFRC_A gRNA1 | GCGCAGCGGGCGTTTTA |
| TFRC_A gRNA2 | TTATAGCCTGGCCCCGC |
| TFRC_B gRNA1 | GCAGCCTCAGAAATACAAAAGTTTT |
| TFRC_B gRNA2 | GATATCGGGTGGCGGCT |
| UCA1 gRNA1 | GGTTTCCTTTTAGATGACGGGTT |
| UCA1 gRNA2 | TGAAAAGAGAGTCAGCGAGTTTTAGA |
| Control GFP gRNA1 | AGCTGGACGGCGACGTA |
| Control GFP gRNA1 | CAGAACACCCCCATCGG |

**Table S8: qRTPCR primers.**

| **Name** | **Sequence** |
| --- | --- |
| MALAT1 Primer set 1 F | GCATTAATTGACAGCTGACCCA |
| MALAT1 Primer set 1 R | GCTTGCTCCTCAGTCCTAGCTT |
| MALAT1 Primer set 2 F | GAGCGAGTGCAATTTGGTGATG |
| MALAT1 Primer set 2 R | ATCCTCTACGCACAACGCC |
| MALAT1 Primer set 3 F | GCCAAATTGAGACAATTTCAGC |
| MALAT1 Primer set 3 R | CGAATTCAGGGTGAGGAAGTA |
| MALAT1 Primer set 4 F | CTGAGTCATAACCAGCCTGGC |
| MALAT1 Primer set 4 R | GCTTATTCCCCAATGGAGGTA |
| TFRC F | AAAATCCGGTGTAGGCACAG |
| TFRC R | GCACTCCAACTGGCAAAGAT |
| UCA1 F | AAAATCTGGGCCAGGGG |
| UCA1 R | CTCTTCACGGAATGAGGGC |
